# Tranilast protects pancreatic β-cells from palmitic acid-induced lipotoxicity via FoxO-1 inhibition

**DOI:** 10.1038/s41598-022-25428-3

**Published:** 2023-01-03

**Authors:** Hye-Eun Choi, Dong Young Kim, Mi Jin Choi, Jea Il Kim, Ok-Hee Kim, Jinwook Lee, Eunhui Seo, Hyae Gyeong Cheon

**Affiliations:** 1 Department of Pharmacology, College of Medicine, Gachon University, Incheon, 21999 Republic of Korea; 2 Department of Health Sciences and Technology, GAIHST, Gachon University, Incheon, 21999 Republic of Korea; 3 College of Pharmacy and Gachon Institute of Pharmaceutical Science, Gachon University, Incheon, 21999 Republic of Korea

**Keywords:** Drug discovery, Endocrinology

## Abstract

Tranilast, an anti-allergic drug used in the treatment of bronchial asthma, was identified as an inhibitor of the transcription factor Forkhead box O-1 (FoxO-1) by high throughput chemical library screening in the present study. Based on FoxO-1’s role in apoptotic cell death and differentiation, we examined the effect of tranilast on palmitic acid (PA)-induced cell damage in INS-1 cells. Tranilast substantially inhibited lipoapoptosis and restored glucose-stimulated insulin secretion under high PA exposure. Moreover, PA-mediated downregulation of PDX-1, MafA, and insulin expression was attenuated by tranilast. PA-induced oxidative and ER stress were also reduced in the presence of tranilast. These protective effects were accompanied by increased phosphorylation and decreased nuclear translocation of FoxO-1. Conversely, the effects of tranilast were diminished when treated in transfected cells with FoxO-1 phosphorylation mutant (S256A), suggesting that the tranilast-mediated effects are associated with inactivation of FoxO-1. Examination of the in vivo effects of tranilast using wild type and diabetic db/db mice showed improved glucose tolerance along with FoxO-1 inactivation in the pancreas of the tranilast-treated groups. Thus, we report here that tranilast has protective effects against PA-induced lipotoxic stress in INS-1 cells, at least partly, via FoxO-1 inactivation, which results in improved glucose tolerance in vivo.

## Introduction

Forkhead box O (FoxO) proteins act as nuclear transcription factors and are involved in negative cross-talk with the insulin signaling pathway^1^. Four isoforms of FoxO (FoxO-1, 3a, 4, and 6) have been identified in mammalian cells, among which FoxO-1 is the most abundant and best studied isoform^2^. Previous studies have shown that overexpression of FoxO-1 in insulin-sensitive tissues is associated with type 2 diabetes mellitus^3^. Of note, FoxO-1 regulates the transcription of two important enzymes involved in hepatic gluconeogenesis (glucose-6 phosphatase and phosphoenolpyruvate carboxykinase)^4,5^. Thus, the regulation of FoxO-1 activity as well as its expression significantly contributes to hepatic glucose production.

β-cell dysfunction is a prominent feature of type 2 diabetes that results in defects in glucose-stimulated insulin secretion^6,7^. Elevation in free fatty acid levels, especially that of saturated fatty acids such as palmitic acid (PA), results in impairment of insulin production and secretion, and β-cell apoptosis^8–10^, which in turn leads to lipotoxicity, serving as a key player in the pathophysiology of type 2 diabetes. Among the various mediators of lipotoxicity, FoxO-1 is known to play a crucial role in β-cell function, inhibiting β-cell replication and neogenesis^11^. Accordingly, FoxO-1 mRNA levels were found to be increased in the islets of patients with type 2 diabetes in clinical studies^3,12^.

FoxO-1 is known to bind to the promoter of pancreatic duodenal homeobox 1 (PDX-1)^13^, a transcription factor involved in β-cell function and survival, leading to the suppression of mRNA expression of insulin, glucagon-like peptide 1, and glucokinase^14^. In addition, several genes involved in apoptosis, and oxidative and ER stress are known to be downstream transcriptional targets of FoxO-1. FoxO-1 activity is regulated through various post-translational modifications, including phosphorylation, acetylation, and ubiquitination. Importantly, FoxO-1 phosphorylation by protein kinase B (PKB/Akt) results in its exclusion from the nucleus, and thus FoxO-1 remains in an inactive state^15^. On the other hand, decreased FoxO-1 phosphorylation allows FoxO-1 to enter the nucleus and play an active role in the transcription of several genes.

Based on the negative role of FoxO-1 in insulin action, FoxO-1 inhibitors may be applicable as a new therapeutic approach to treat metabolic disorders, including type 2 diabetes. However, FoxO-1 as a potential therapeutic target remains underutilized due to the non-availability of specific and potent inhibitors. To identify new FoxO-1 inhibitors, we screened a chemical library of the Korean Chemical Bank for FoxO-1 inhibitory activity, and identified tranilast as a FoxO-1 inhibitor.

Tranilast (N-[3’,4’-dimethoxycinnamoyl]-anthranilic acid) is an analog of a tryptophan metabolite^16^. Initially, tranilast was developed as an anti-allergic agent and is currently used for the treatment of inflammatory diseases such as bronchial asthma, atypical dermatitis, and allergic conjunctivitis^17^. Its main mechanism of action is attributed to its inhibitory action on the release of the chemical mediator, histamine^18^. In the present study, the pharmacological profile of tranilast as a FoxO-1 inhibitor was investigated for its repositioning as a potential anti-diabetic agent with special focus on β-cell function. Since lipotoxicity induced by saturated fatty acids appears to be an important cause of impairment of β-cell function^19^, PA-induced lipotoxicity model was employed in the present study, and the effect of tranilast on rat INS-1 pancreatic β-cells following PA exposure was examined. The in vivo efficacy of tranilast was evaluated using two mouse strains, C57BL/6J and db/db, based on the facts that C57BL/6J strain is the popular stain for metabolic research with diabetes prone characteristics, and db/db strain is a well-known type 2 diabetes animal model with β-cell dysfunction.

## Results

### Tranilast inhibits FoxO-1 activity

**Figure 1:**
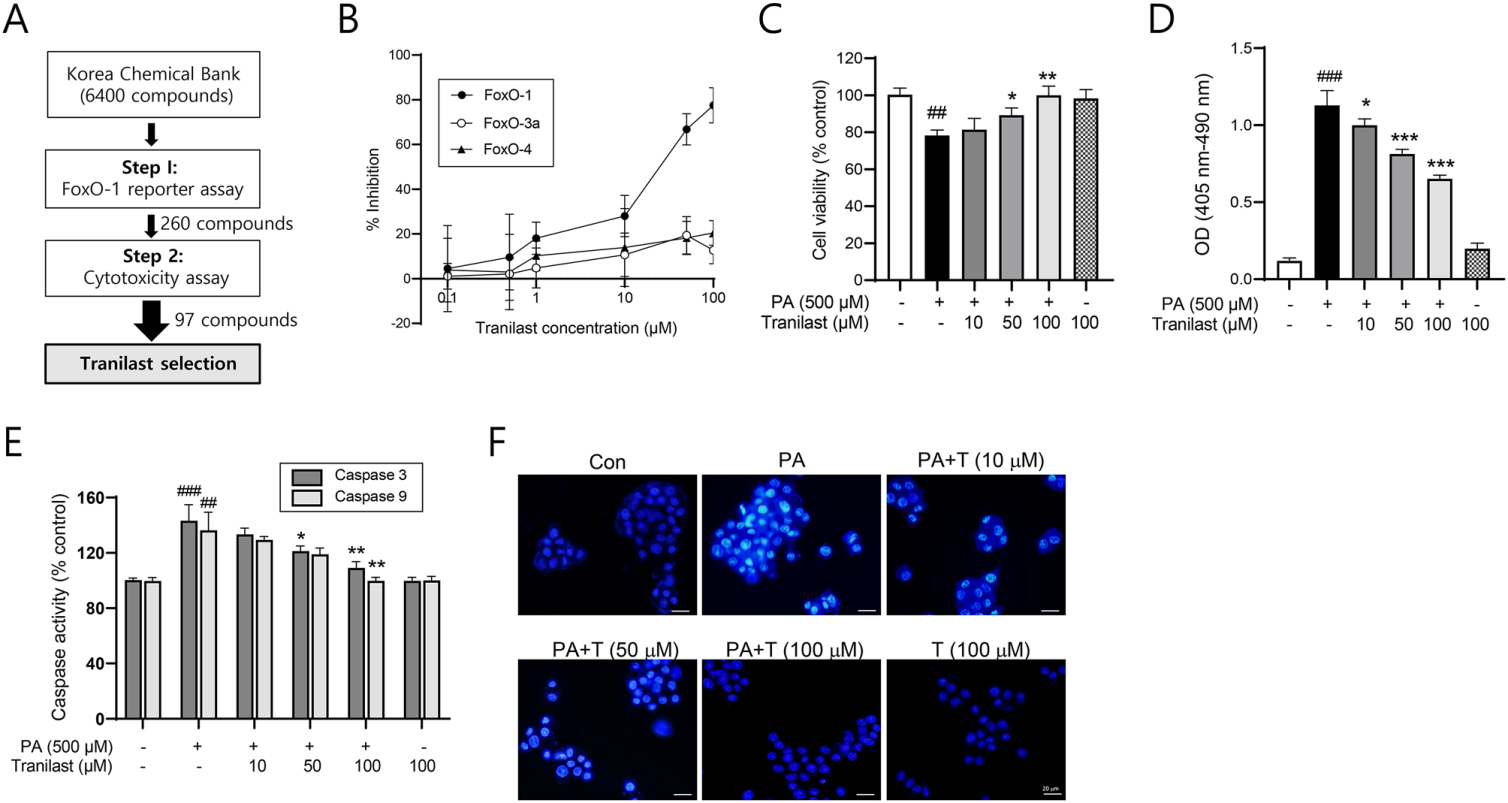
Tranilast protects INS-1 cells from PA-induced cell death. (**A**) Schematic representation of tranilast selection from high throughput screening of Korea Chemical Bank. (**B**) Inhibitory effects of tranilast on FoxO-1, 3a, and 4 were determined using HepG2 cells after transfection with FoxO-1 vector, and IRE vector as described in Methods. (**C**) Effect of tranilast on cell viability was measured using MTT assay following incubation of INS-1 cells with PA (500 μM) for 24 h in the presence or absence of tranilast at the indicated concentrations. (**D**) INS-1 cells were treated with PA (500 μM) for 24 h in the presence or absence of various concentrations of tranilast. Induction of apoptosis was measured using a cell death detection ELISA kit. (**E**) Caspase-3 and -9 activities were measured using the chromogenic substrates Ac-DEVD-pNA and Ac-LEHD-pNA, respectively. (**F**) DAPI staining of INS-1 cells following incubation with PA and tranilast. Representative results are shown (scale bar = 20 μm). Data are expressed as mean ± standard deviation (SD) of three independent experiments, each done in duplicate. ##*p* < 0.01, ###*p* < 0.005 versus control, *p < 0.05, **p < 0.01, ***p < 0.005 versus PA alone.

We conducted high throughput chemical library screening using the Korea Chemical Bank (6,400 compounds tested at 50 μM) to identify new FoxO-1 inhibitors, and initially identified 260 active hits. 163 hits were excluded for further study due to their cytotoxicity, and 97 hits were confirmed. Among those, tranilast was selected for the biochemical and pharmacological characterization as a FoxO-1 inhibitor (Fig. 1A). To confirm the FoxO-1 inhibitory effect of tranilast, a FoxO-1 reporter assay was carried out in the presence of various concentrations of tranilast. In HepG2 cells transiently transfected with FoxO-1 expression vector and IRE promoter construct, tranilast inhibited FoxO-1 activity in a concentration-dependent manner, with an estimated IC_50_ of 30 μM (Fig. 1B). In contrast, the effect of tranilast on FoxO-3a and FoxO-4 appeared to be minimal, showing that tranilast weakly inhibited FoxO-3a and FoxO-4 promoter-driven activity (12.7 ± 6.06% and 20.5 ± 5.35% inhibition at 100 μM, respectively) (Fig. 1B). These results suggest that tranilast exhibits moderate selectivity for FoxO-1 relative to the other FoxO isoforms.

### Tranilast reduces PA-induced apoptosis in INS-1 cells

The role of FoxO-1 in stress-induced cell death appears to be context- and cell type-dependent. Therefore, we investigated the effect of tranilast on PA-induced apoptotic cell death in INS-1 cells. Cell viability was reduced by 21.7 ± 3.65% following PA exposure (500 μM) for 24 h, which was restored in the presence of tranilast in a concentration-dependent manner. Complete restoration of cell viability was achieved following treatment with 100 μM tranilast (Fig. 1C). To examine the anti-apoptotic effect of tranilast, the extent of cell apoptosis was determined in various assays. First, the apoptotic rate was measured photometrically based on peroxidase activity. PA-induced apoptotic death was significantly and concentration dependently reduced following treatment with tranilast (Fig. 1D). In addition, PA-induced increase in caspase-3/9 activity was decreased by tranilast treatment (Fig. 1E). The anti-apoptotic effect of tranilast was further confirmed by DAPI staining (Fig. 1F), where reduced DNA fragmentation and reduced nuclear permeability and morphological changes were observed following tranilast treatment.

**Figure 2:**
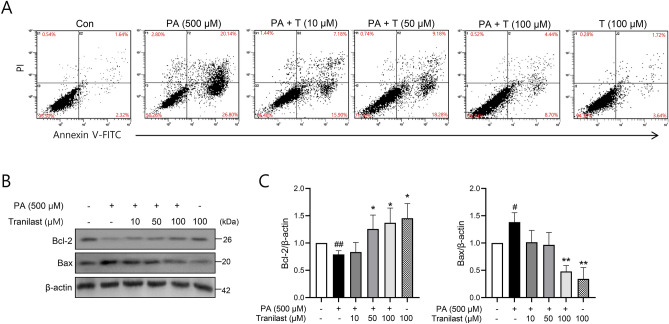
Tranilast reduces PA-induced apoptosis in INS-1 cells. (**A**) INS-1 cells were treated with PA (500 μM) in the presence or absence of tranilast at the indicated concentrations. The apoptotic rate of INS-1 cells treated with PA and tranilast was determined by flow cytometry. Representative results are shown. (**B**) Bcl-2 and Bax protein levels were determined by western blot analysis, and standardized to the expression of β-actin. Original scans of whole blots are shown in Supplementary information. **(C)** Density analyses of western blots were shown. Data are expressed as mean ± SD of three independent experiments, each done in duplicate. #*p* < 0.05, ##*p* < 0.01 versus control, *p < 0.05, **p < 0.01vs. PA alone.

Next, we assessed the anti-apoptotic effect of tranilast using flow cytometric analysis. The results showed that PA markedly increased apoptotic cell death (46.9%), which was reduced following tranilast treatment (13.1% at 100 μM) (Fig. 2A). In addition, following treatment of the cells with tranilast, we observed an increase in the anti-apoptotic, Bcl-2, and a decrease in the pro-apoptotic Bax levels compared to that in the control group (Fig. 2B and C). These results suggest that tranilast protects INS-1 cells from PA-induced cell damage by inhibiting apoptotic cell death.

### Tranilast reduces PA-induced oxidative stress and ER stress in INS-1 cells

**Figure 3:**
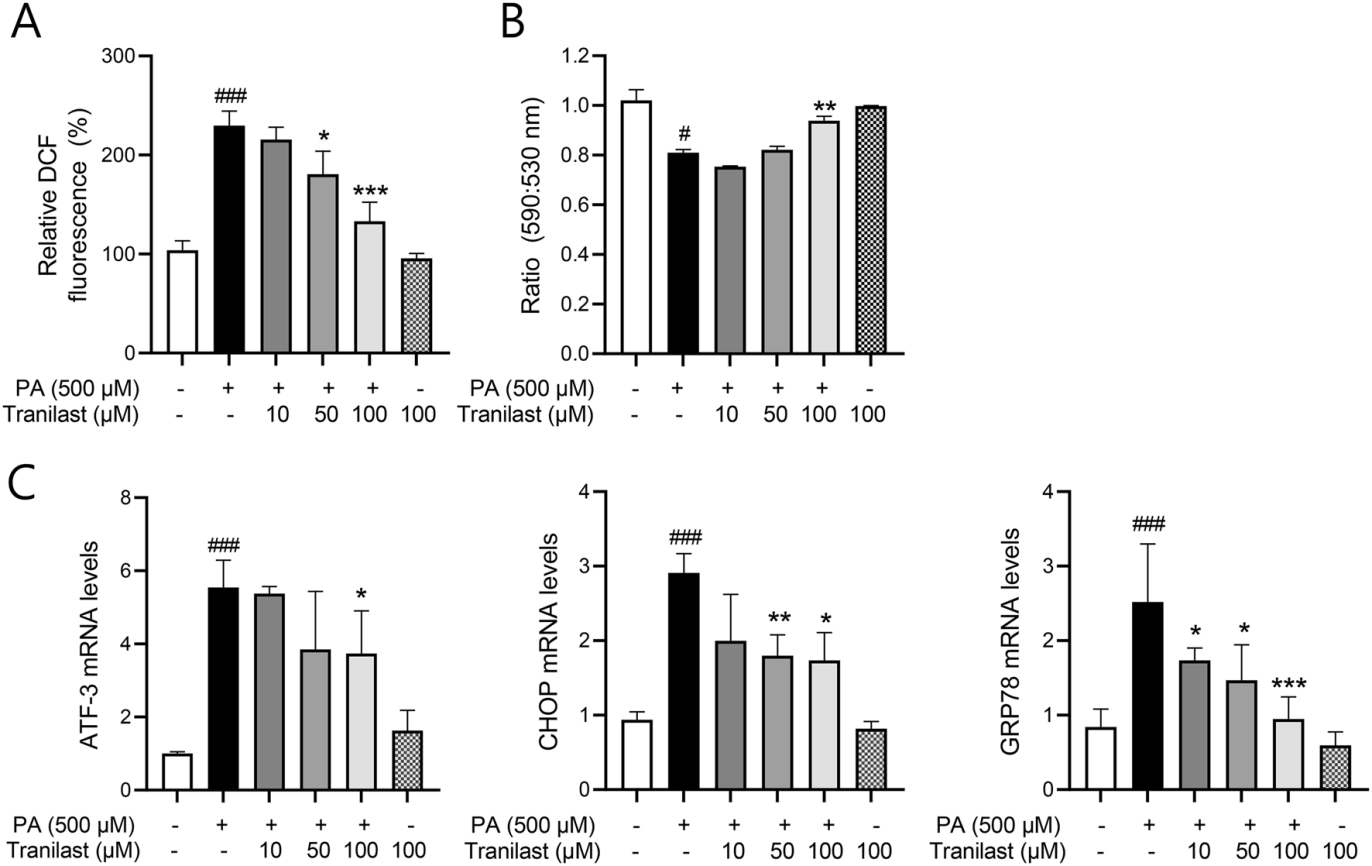
Tranilast reduces PA-induced oxidative and ER stress in INS-1 cells. (**A**) INS-1 cells were treated with PA (500 μM) in the presence or absence of tranilast at the indicated concentrations. Intracellular ROS levels in the treated-cell lysates were detected by DCF fluorescence using a colorimetric assay kit as described in Methods. (**B**) Treated cells were stained with JC-1 probe and analyzed using a fluorescence spectrophotometer. (**C**) mRNA expression of ER stress markers was determined by real-time qPCR. Data are expressed as mean ± SD of three independent experiments, each done in duplicate. #*p* < 0.05, ###*p* < 0.005 versus control, *p < 0.05, **p < 0.01, ***p < 0.005 versus PA alone.

As β-cells are sensitive to both oxidative and ER stress, we examined the effect of tranilast on PA-induced oxidative and ER stress. First, the effect of tranilast on PA-induced ROS production was determined using a DCF-DA probe. As shown in Fig. 3A, intracellular ROS levels were elevated to 226 ± 20.0% following PA exposure as compared to the control, which was significantly attenuated in the presence of tranilast in a concentration-dependent manner (132 ± 12.1% at 100 μM, *p* < 0.005).

Oxidative stress results in mitochondrial dysfunction reflected as changes in the mitochondrial transmembrane potential. Here, JC-1, a dye for monitoring mitochondrial membrane potential, was used to examine the effect of tranilast on mitochondrial function. PA induced the breakdown of mitochondrial membrane potential by 19.3 ± 1.75%, which was restored almost to normal levels by treatment with 100 μM tranilast (Fig. 3B). Furthermore, PA-induced mRNA expression of ER stress markers was also mitigated following tranilast treatment (Fig. 3C), suggesting that tranilast has protective effects against PA-induced oxidative and ER stress.

### Tranilast restores PA-induced functional impairment of β-cells

**Figure 4:**
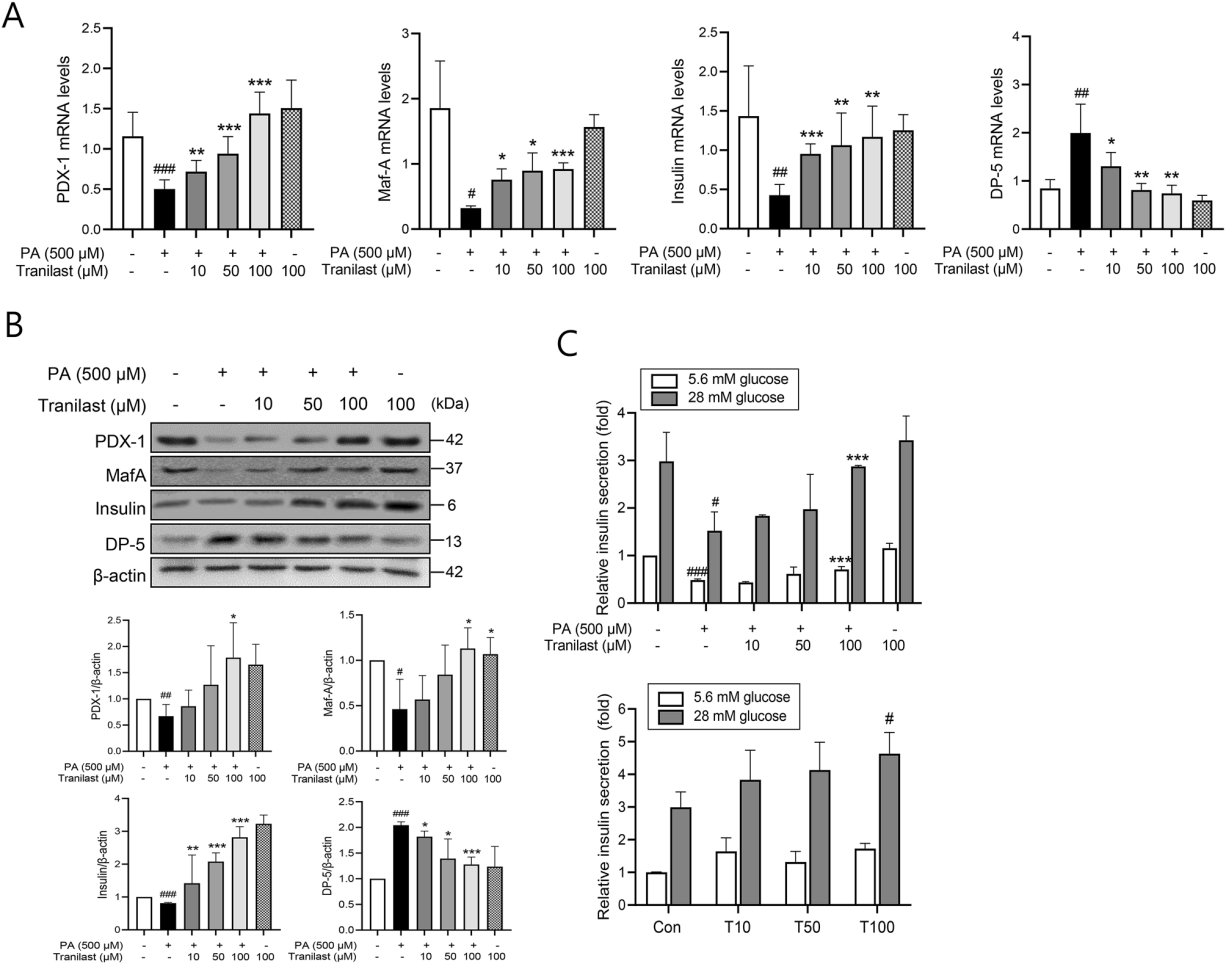
Tranilast restores PA-induced functional impairment. (**A,B**) INS-1 cells were treated with PA (500 μM) in the presence or absence of tranilast at the indicated concentrations for 8 h (real-time qPCR) or 24 h (western blotting). Expression of PDX-1, MafA, insulin, and DP-5 was determined by real-time qPCR **(A)**, and by western blot analysis **(B)**. Original scans of whole blots are shown in Supplementary information. Density analyses of western blots were shown in the lower panel. Data are expressed as mean ± SD of three independent experiments, each done in duplicate. #*p* < 0.05, ##*p* < 0.01, ###*p* < 0.005 versus control, *p < 0.05, **p < 0.01, ***p < 0.005 versus PA alone. **(C** upper**)** INS-1 cells were treated with PA (500 μM) in the presence or absence of tranilast at the indicated concentrations for 24 h, and further incubated in the same buffer containing 5.6 or 28 mM glucose for 30 min. Insulin level in the media was quantified using an insulin RIA kit. **(C** lower**)** Primary islets isolated from C57BL/6J mice were treated with various concentrations of tranilast for 24 h, and the insulin content in the media was measured using mouse insulin RIA kit. Data are expressed as mean ± SD of three independent experiments, each done in duplicate. #*p* < 0.05, ###*p* < 0.005 versus control, ***p < 0.005 versus PA alone.

Both PDX-1 and MafA, that are essential for the maintenance of normal β-cell function, are transcriptionally regulated by FoxO-1. Real-time qPCR revealed that the mRNA expression of PDX-1, MafA, and insulin were reduced by PA, whereas their expression was restored to control cell levels by tranilast. In contrast, the mRNA expression of DP-5 (a cell death promoting gene) was markedly increased by PA, whereas the level was restored by tranilast (Fig. 4A). The effect of tranilast on the mRNA expression of these markers was also confirmed at the protein level, as shown in Fig. 4B.

To further investigate the effect of tranilast on β-cell function, we assessed insulin secretion in response to glucose stimulation. As expected, PA significantly impaired GSIS in INS-1 cells (3.0 ± 0.6-fold in control vs. 1.5 ± 0.3-fold in PA treated group at 28 mM glucose, *p* < 0.05), which was restored by tranilast (Fig. 4C upper). Additionally, when primary islets isolated from C57BL/6J mice were treated with tranilast, increased GSIS was observed (2.9 ± 0.6-fold in control vs. 4.7 ± 0.9-fold in 100 μM tranilast-treated group, *p* < 0.05) (Fig. 4C lower). These data showed that tranilast enhanced insulin release in primary islets, and effectively restored PA-mediated GSIS impairment. Taken together, these results demonstrated that the PA-induced functional impairments of β-cells are ameliorated by tranilast treatment, consistent with the effect of tranilast on the mRNA and protein expression of β-cell neogenesis and survival-related genes.

### Protective effects of tranilast are associated with FoxO-1 inactivation

**Figure 5:**
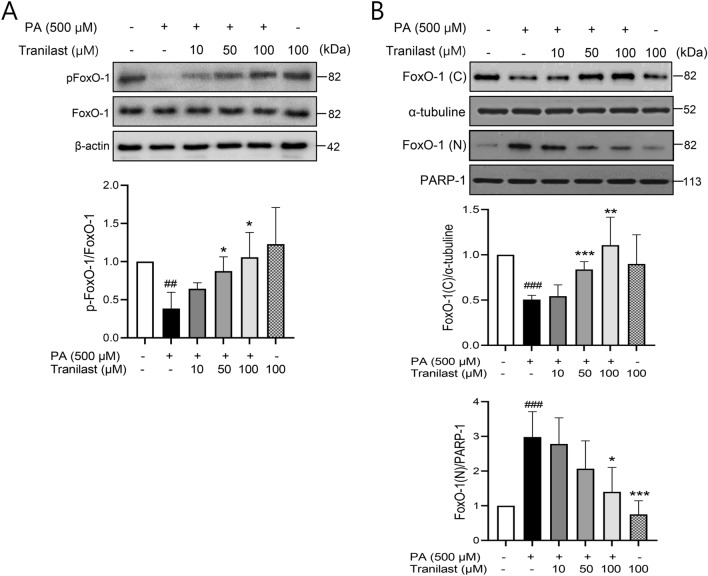
Protective effects of tranilast are associated with FoxO-1 inactivation. (**A,B**) INS-1 cells were treated with PA (500 μM) in the presence or absence of tranilast at the indicated concentrations for 24 h, and immunoblotting of whole cell lysates **(A)**, or subcellular fractions (**B**, cytoplasmic and nucleus fractions) were carried out. Representative blots of three independent experiments are shown, and density analyses of western blots were presented in the lower panel. Original scans of whole blots are shown in Supplementary information. ##*p* < 0.01, ###*p* < 0.005 versus control, *p < 0.05, **p < 0.01, ***p < 0.005 versus PA alone.

To elucidate the underlying mechanism of action of tranilast with a special focus on FoxO-1, the levels of phosphorylated FoxO-1 (pFoxO-1) were assessed following PA and tranilast treatments. Compared to the control group, PA treatment significantly reduced the level of pFoxO-1 in whole cell lysates (Fig. 5A), and this decrease was accompanied by increased nuclear translocation of FoxO-1 (Fig. 5B), indicating that PA led to FoxO-1 activation. FoxO-1 is known to shuttle between the nucleus and cytoplasm, and its location affects its transcriptional activity. To determine whether tranilast alters pFoxO-1 levels and subsequent FoxO-1 localization, the nuclear and cytoplasmic fractions were subjected to western blot analysis. Consistent with the inhibitory effect of tranilast on FoxO-1 (Fig. 1A), tranilast increased pFoxO-1 in whole cell lysate with concurrent reduction of nuclear FoxO-1 levels (Fig. 5A and B), indicating that tranilast inhibited PA-induced FoxO-1 activation. These findings suggest that the protective action of tranilast against PA-induced β-cell lipotoxicity is associated with FoxO-1 inactivation.

**Figure 6:**
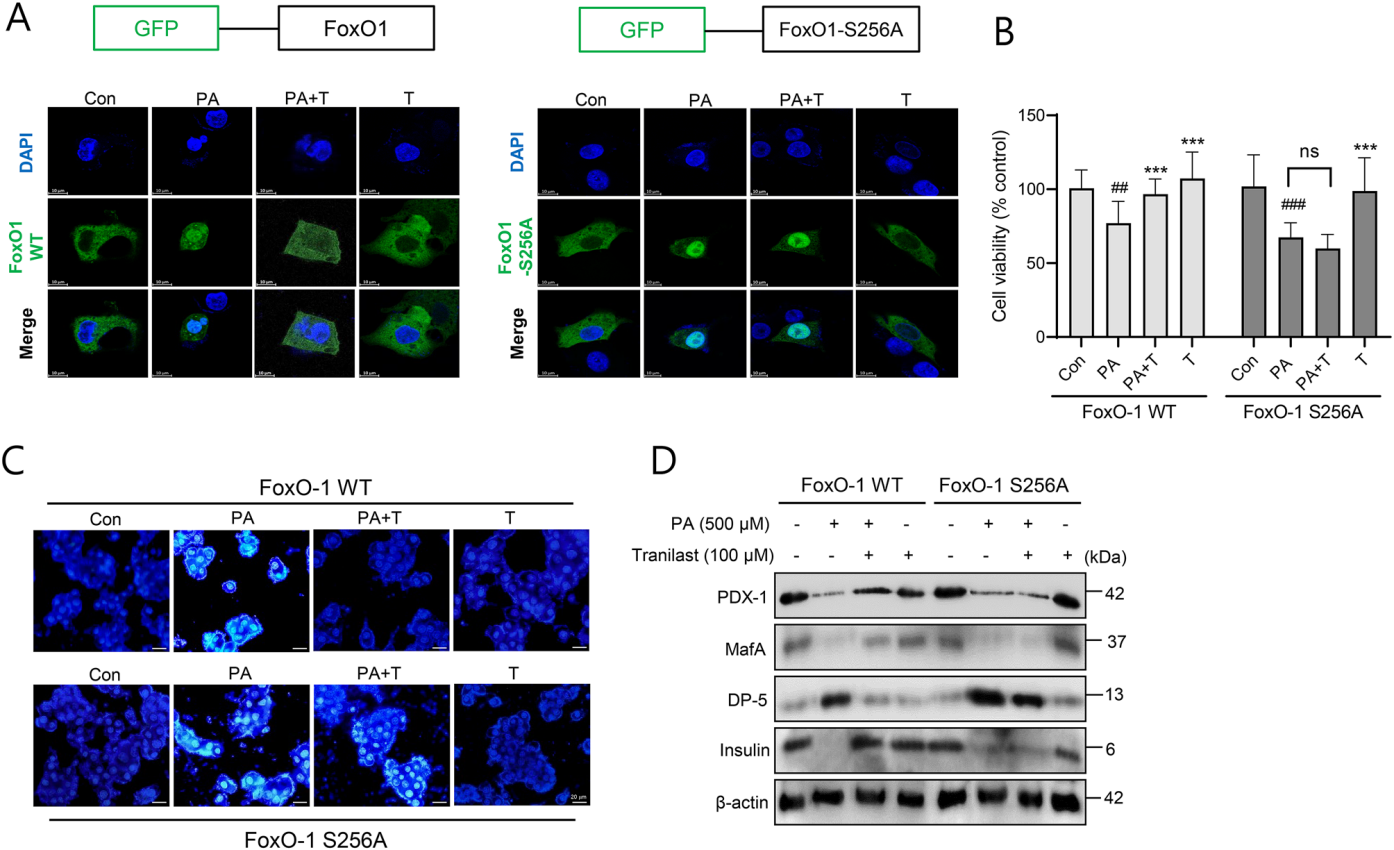
Effects of FoxO-1 S256A mutant on tranilast action. (**A**) Either FoxO-1 wild type (WT) or FoxO-1 mutant (S256A) was transfected into CHO cells, and the FoxO-1 localization after tranilast (100 μM) and PA (500 μM) treatment was observed using confocal microscopy. **(B–D)** INS-1 cells were transfected with either FoxO-1 WT or FoxO-1 mutant, and then treated with tranilast (100 μM) in the presence of PA for 24 h. Cell viability **(B)**, DAPI staining **(C)** and western blot **(D)** were determined (scale bar = 20 μm). Original scans of whole blots are shown in Supplementary information. Data are expressed as mean ± SD of three independent experiments, each done in duplicate **(B)**. Representative results are shown **(A,C,D)**. ##*p* < 0.01, ###*p* < 0.005 versus control, ***p < 0.005 versus PA alone.

Next, we determined the effects of tranilast after the cells were transfected with FoxO-1 phosphorylation mutant (substituted ser256 to ala; S256A) to further confirm that tranilast actions are attributed to the FoxO-1 phosphorylation. As expected, the nuclear FoxO-1 induced by PA was translocated to the cytoplasm by tranilast treatment in wild type FoxO-1 transfected cells, whereas tranilast-induced FoxO-1 translocation from the nucleus to the cytoplasm was disappeared in FoxO-1 S256A mutant-transfected cells (Fig. 6A). Correspondingly, protective effects of tranilast from PA-induced apoptotic cell death were abolished in FoxO-1 S256A mutant-transfected cells (Fig. 6B and C). The effects of tranilast on the FoxO-1 target protein levels were also diminished by FoxO-1 S256A transfection (Fig. 6D). These results confirm that tranilast inactivated FoxO-1 via increased its phosphorylation, thereby offering beneficial effects against PA-induced lipotoxicity in INS-1 cells.

### Tranilast improves glucose intolerance in vivo

**Figure 7:**
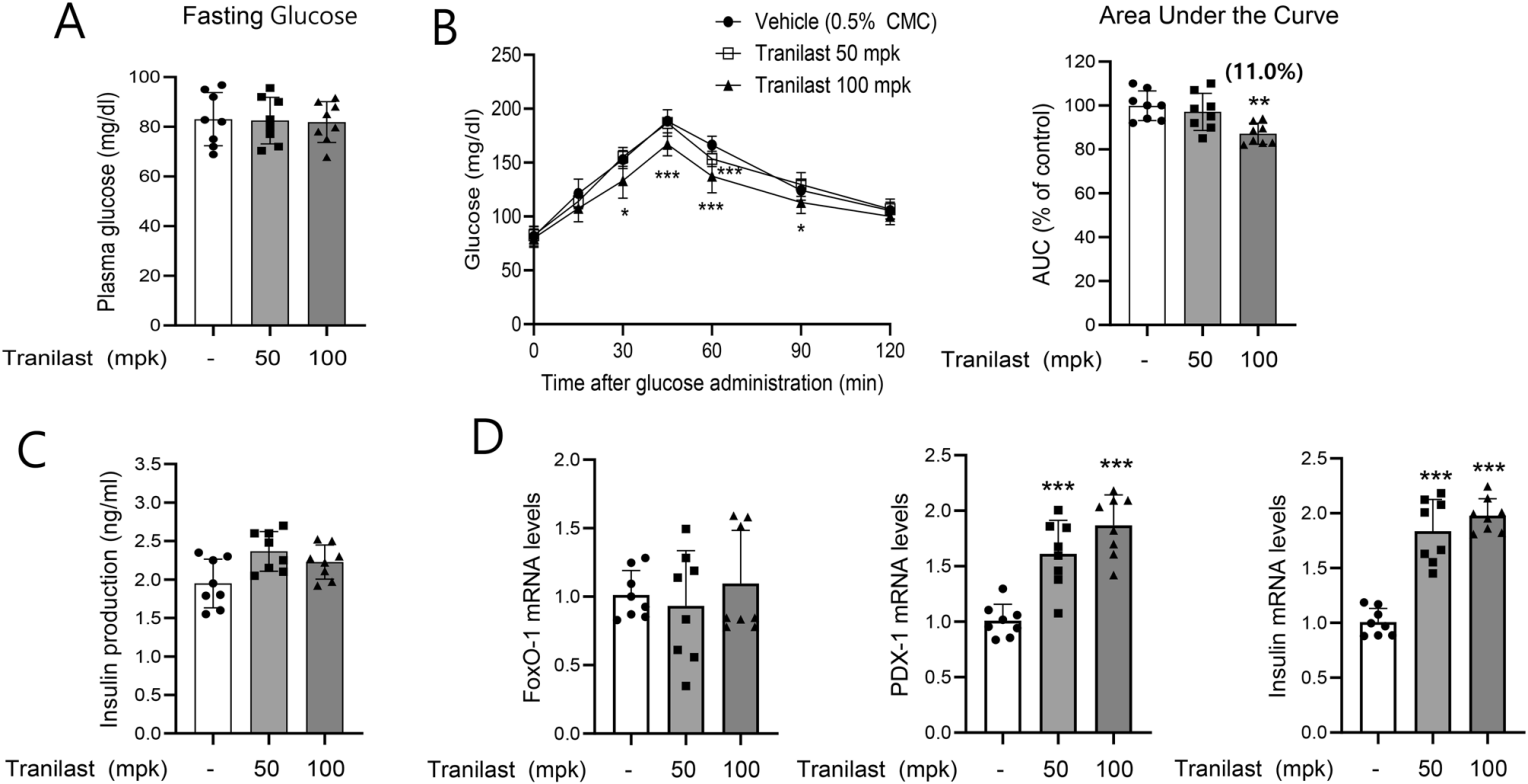
Acute in vivo effect of tranilast was studied using C57BL/6J mice. Male C57BL/6J mice (6-weeks old) were orally administered either vehicle (0.5% CMC) or tranilast (50 and 100 mg/kg, n = 8 per group) at three time points (9 AM, 7 PM, and 9 AM on the 2nd day). **(A,C)** Fasting glucose and insulin levels were determined after overnight fasting, and glucose tolerance test was carried out **(B)**. **(D)** mRNA levels of PDX-1, insulin, and FoxO-1 in whole pancreas were measured by real-time qPCR. *p < 0.05, **p < 0.01, ***p < 0.005 versus vehicle group.

Tranilast (50 and 100 mg/kg) was orally administered to C57BL/6J mice three times over 2 days, and a glucose tolerance test was performed. As expected, no difference was observed in fasting plasma glucose levels between vehicle and tranilast-treated groups, but tranilast improved glucose clearance compared to the vehicle group (Fig. 7A and B). No significant difference in plasma insulin levels was also noted (Fig. 7C). Consistent with the in vitro results, tranilast administration induced mRNA expression of insulin and PDX-1 in the pancreas, but no alteration in FoxO-1 mRNA expression was detected (Fig. 7D).

**Figure 8:**
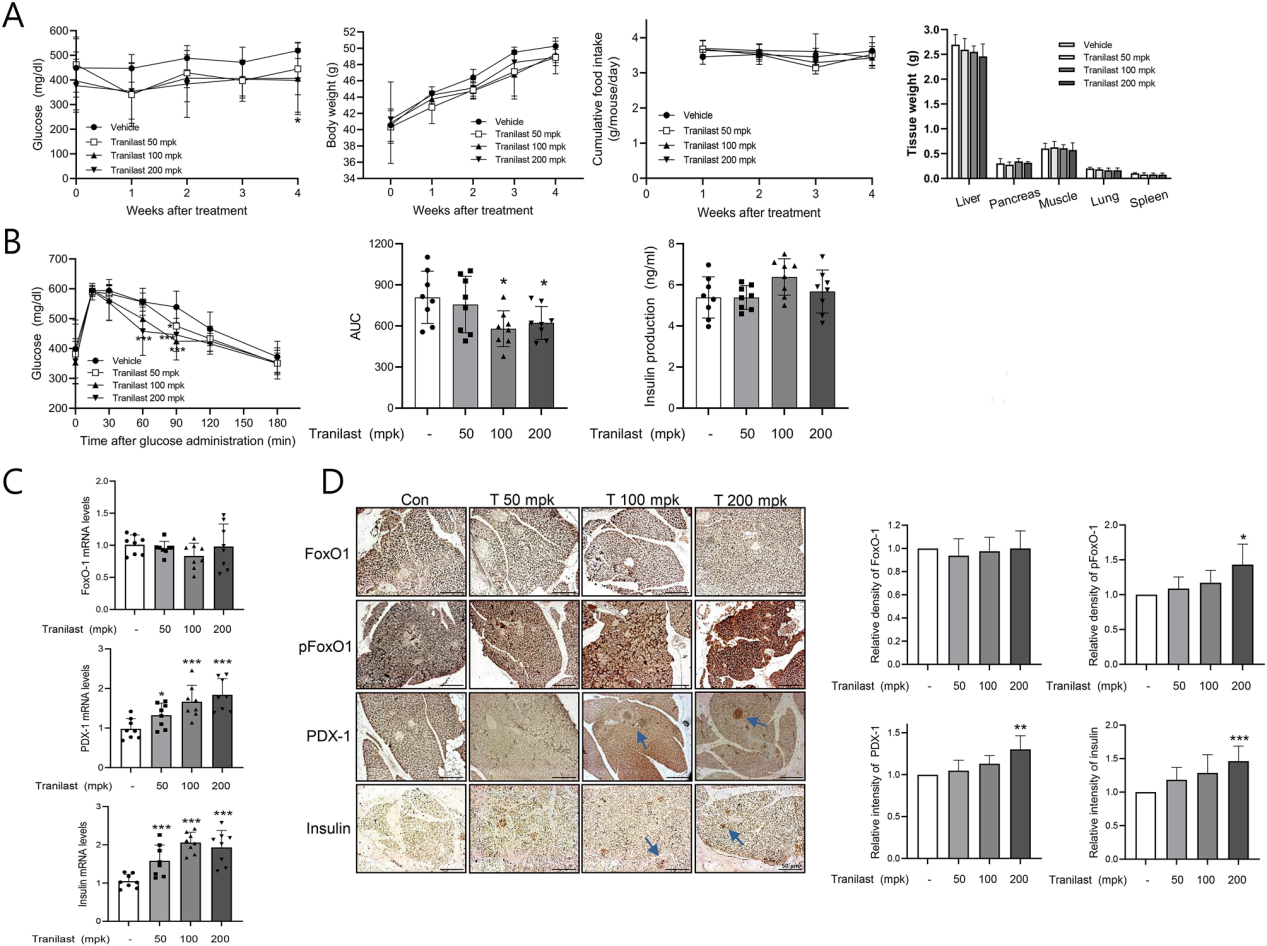
Chronic in vivo effect of tranilast was studied using db/db mice. Male db/db mice (8-weeks old) were orally administered either vehicle (0.5% CMC) or tranilast (50, 100, and 200 mg/kg) once daily for 4 weeks (n = 8 per group). **(A)** Non-fasting blood glucose, body weight and food intake were determined once a week. At the end of treatment, tissues were isolated and weighed. **(B)** After overnight fasting, oral glucose tolerance test was carried out after 4 weeks of tranilast administration. Area under the curve and fasting plasma insulin levels were determined. **(C)** mRNA levels of PDX-1, insulin, and FoxO-1 in whole pancreas were measured by real-time qPCR. **(D)** Representative pancreatic sections with immunohistochemical staining of pFoxO-1, FoxO-1, PDX-1, and insulin are shown (scale bar = 50 μm). The results of quantitative analysis were shown in the right panel. *p < 0.05, **p < 0.01, ***p < 0.005 versus vehicle group.

The in vivo effects of tranilast were further investigated in db/db mice after oral administration of tranilast (50, 100, 200 mg/kg) for 4 weeks. The levels of non-fasting blood glucose decreased in the tranilast-treated group (Fig. 8A), with improved glucose tolerance as determined by a glucose tolerance test (Fig. 8B). The effects of tranilast on body weight, tissue weight and food intake were unnoticeable (Fig. 8A). Although the expression of insulin in the pancreas was increased in the tranilast-treated group (Fig. 8C and D), plasma insulin levels were little affected by tranilast (Fig. 8B), possibly due to the hyperinsulinemia already present in db/db mice. As expected, the mRNA level of PDX-1 was increased in the pancreas of the tranilast-treated groups, with little difference in FoxO-1 mRNA levels (Fig. 8C). Correspondingly, immunohistochemical staining showed that the pancreatic tissues exhibited stronger expression of PDX-1 in the tranilast-treated groups than those in the vehicle-treated group (Fig. 8D), which was consistent with the in vitro results. The levels of pFoxO-1 in the pancreatic tissues were also markedly increased in tranilast-treated pancreatic tissues compared to the vehicle-treated pancreatic tissues with little change in FoxO-1 levels (Fig. 8D). These in vivo results further suggest that tranilast improves glucose tolerance in vivo, possibly via FoxO-1 inactivation in the pancreatic β-cells.

## Discussion

In this study, we investigated the protective effect of tranilast on PA-induced β-cell dysfunction, and its mechanism of action as a FoxO-1 inhibitor using INS-1 cells. We found that tranilast ameliorated PA-induced β-cell lipotoxicity via FoxO-1 inhibition in vitro. In vivo administration of tranilast improved glucose tolerance with increased pFoxO-1 and PDX-1 levels in the pancreas of db/db mice. These data suggest the possible repositioning of tranilast as an anti-diabetic agent.

Normal pancreatic β-cell function and mass are critical for maintaining glucose homeostasis, and any defect significantly contributes to the development of diabetes mellitus^7^. Various genetic and environmental factors are implicated in β-cell dysfunction, and most notably chronic exposure to saturated fatty acids induces β-cell dysfunction and apoptosis, leading to the development of type 2 diabetes^10^. Thus, protection of β-cells from saturated fatty acid-induced damage is critically important for preventing impairment of glucose homeostasis. The detailed molecular mechanisms involved in PA-induced β-cell dysfunction and apoptosis have not been fully elucidated, although several pathways such as those mediating mitochondrial dysfunction, ER stress, and oxidative stress have been implicated^20–22^. Consistently, we found that PA (500 μM) induced mitochondrial dysfunction and ER and oxidative stress leading to INS-1 β-cell dysfunction and apoptosis in our study.

Among the many factors involved in β-cell maintenance, FoxO-1 plays a key regulatory role in β-cell mass and function^23,24^. FoxO-1 is a negative regulator of the transcription factor, PDX-1 that is crucial for β-cell growth and function^25^. FoxO-1 inactivation leads to increased PDX-1 expression and β-cell proliferation, and FoxO-1 suppression reduces the expression of apoptotic markers^11^. In contrast, FoxO-1 overexpression promotes β-cell apoptosis by reducing CD24 expression^26^. In addition to its role in β-cell apoptosis, FoxO-1 activity also affects GSIS and modulates the transcription of FoxO-1 target genes including PDX-1, MafA, and insulin in β-cells. In agreement with the role of FoxO-1 in β-cells, tranilast prevented PA-induced apoptosis via increased PDX-1 transcription in our study, possibly acting as a FoxO-1 inhibitor.

Accumulating evidence suggests that oxidative stress also contributes to structural and functional damage of β-cells^27^. Interestingly, FoxO-1 activity is influenced by oxidative stress through various post-translational modifications, such as phosphorylation, acetylation, and ubiquitination^28^. For example, FoxO-1 phosphorylation in β-cells is reduced by oxidative stress, allowing its translocation from the cytoplasm to the nucleus^28,29^. In contrast, inhibition of PDX-1 expression in response to lipotoxicity due to oxidative stress, results in its increased translocation from the nucleus to the cytoplasm^30^. Accordingly, our results showed that tranilast treatment attenuated PA-induced oxidative stress, in association with increased shuttling of FoxO-1 from the nucleus to the cytoplasm.

In the present study, we uncovered a novel mechanism of action of tranilast and demonstrated its protective effects against PA-induced β-cell lipotoxicity. Tranilast inhibited PA-induced apoptosis in a concentration-dependent manner. Consistent with the findings that mitochondrial depolarization induced by PA leads to β-cell apoptosis^31^, tranilast relieved mitochondrial depolarization and upregulated the Bcl-2/Bax ratio, thereby restoring PA-induced mitochondrial dysfunction. In addition, ER stress in response to lipotoxicity was shown to contribute to β-cell apoptosis^11^, which was mitigated by tranilast treatment, thus adding to the protective mechanisms against PA-induced β-cell damage. Consistent with our results, FoxO-1 inhibition downregulated the expression of ER stress markers and promoted β-cell survival under free fatty acid stress^11^. Pancreatic β-cells are highly susceptible to oxidative stress due to their poor antioxidant defense mechanisms^32^. Our current data revealed that PA-induced ROS production was also inhibited following treatment with tranilast.

The role of FoxO-1 inactivation in tranilast activity was confirmed through many experiments. In agreement with the inhibitory effect of tranilast on FoxO-1 activity observed using the FoxO-1 reporter assay, the expression of downstream targets of FoxO-1 was also altered by tranilast. For example, the pro-apoptotic Bcl-2 family member Bax, PDX-1, MafA, and insulin are known FoxO-1 target genes^29^. Real-time qPCR revealed that the mRNA levels of these FoxO-1 target genes were regulated by PA and corrected by tranilast treatment. Interestingly, increased phosphorylation of FoxO-1 was observed in the presence of tranilast that was accompanied by reduction of nuclear levels of FoxO-1. Moreover, the protective effects of tranilast from PA-induced lipotoxicity were abrogated when the cells were transfected with FoxO-1 S256A phosphorylation mutant, in association with the failure of FoxO-1translocation from the nucleus to the cytoplasm. These findings further support that tranilast inhibits FoxO-1 at least partly via increased its phosphorylation.

PI3K/Akt signaling plays a central role in β-cell growth and function^33–35^. Furthermore, this signaling pathway is involved in the regulation of FoxO-1 transcriptional activity^8^. In contrast, activation of the JNK signaling pathway in response to oxidative stress leads to inhibition of Akt and subsequent dephosphorylation of FoxO-1, which results in nuclear accumulation of FoxO-1^29,36^. Interestingly, we found that increased pFoxO-1 following tranilast treatment was accompanied by decreased Akt phosphorylation, but increased JNK phosphorylation (results not shown). Thus, besides its inhibitory activity on FoxO-1, tranilast may function via additional mechanisms of action, such as by regulation of PI3K/Akt/JNK phosphorylation. The detailed mechanism of tranilast-mediated induction of FoxO-1 phosphorylation needs to be elucidated further.

In vivo studies were carried out to examine the efficacy of tranilast using C57BL/6J and db/db mice. Improved glucose tolerance was observed following tranilast administration in both strains, with increased expression of PDX-1 and insulin, possibly via FoxO-1 inactivation in pancreatic β-cells. Other mechanisms of action of tranilast (i.e., anti-inflammatory effects) may also contribute to the improved glucose tolerance in vivo. On the other hand, relatively mild in vivo effects of tranilast were observed, which may be attributed to several factors including the pharmacokinetic properties of tranilast and the mouse conditions (normal C57BL/6J mice; 7–11 weeks old db/db mice when β cell mass was moderately increased). Although we focused on the FoxO-1 inhibitory effect of tranilast in pancreatic β-cells in the present study, other insulin-sensitive tissues such as the liver may also be affected by tranilast-mediated FoxO-1 inhibition. In summary, we found that tranilast protected pancreatic β-cells from PA-induced lipotoxicity and improved glucose tolerance at least partly through FoxO-1 inactivation. Notably, tranilast shows limited adverse effects and is well tolerated in patients. Based on the current study, tranilast may be of additional therapeutic value in the treatment of metabolic disorders such as type 2 diabetes mellitus.

## Methods

### Cell culture

Rat INS-1 pancreatic β-cells obtained from the Korean Cell Line Bank (Seoul, Korea) were grown in a cell culture flask in 12 mL of RPMI medium containing 10% FBS, 1% penicillin, and 1% streptomycin at 37 °C in an atmosphere of 5% CO_2_ and 95% air. For the purpose of the present study, batches of cells were plated in 24-well plates at a density of 2 × 10^5^ cells/well and cultured for 24 h in RPMI medium. Then, the cells were incubated with various concentrations (10, 50, or 100 µM) of tranilast for 1 h, followed by PA (500 μM) treatment for the indicated times. Bovine serum albumin (BSA)-bound PA in ethanol was prepared as described previously^37^.

### FoxO luciferase reporter assay and high throughput screening using the Korea Chemical Bank

To discover novel FoxO-1 inhibitors, FoxO-1 luciferase reporter assay was established. HepG2 cells obtained from the Korean Cell Line Bank (3 × 10^6^ cells) were transfected with FoxO-1 vector (1098 pcDNA-GFP-FKHR; Addgene, Watertown, MA, USA), and IRE vector (pGL3-4xIRE-Luc), and then seeded into 96 well plates at a density of 2 × 10^4^ cells/well. After 24 h incubation with compounds (50 μM in DMSO), luciferase activity was measured using the Dual Luciferase Reporter Assay Kit (Promega, Fitchburg, WI, USA), and normalized to Renilla luciferase activity for each compound.

The effect of tranilast on FoxO-1 transcriptional activity was confirmed as previously described^38^. Briefly, HepG2 cells were plated at 1 × 10^4^ cells/well in a 96-well plate and cultured for 24 h. The medium was replaced with serum-free, antibiotic-free medium immediately before transfection. X-tream, FoxO-1 vector and IRE vector were mixed with each other at a concentration of 50 ng/μL in serum-free, antibiotic-free medium and allowed to stand at room temperature for 15 min. After 24 h of transfection, the medium was replaced with fresh medium supplemented with 10% FBS and 1% penicillin/streptomycin and treated with various concentrations of tranilast for 24 h, and then luciferase activity was measured. FoxO-3a and FoxO-4 transcriptional activities were determined in a similar manner, except that the specific expression vector for each of these isoforms was used in the transfection.

### MTT assay for cell viability

INS-1 cells were plated at a density of 1 × 10^4^ cells/well in 96-well plates. Cell viability was measured after treatment of cells with or without PA (500 μM) in the absence or presence of various concentrations (10, 50, or 100 µM) of tranilast (1 h pretreatment) for 24 h. Then, the cells were incubated with MTT solution for 4 h at 37 °C under 5% CO_2_. DMSO (200 μL) was added to extract the MTT formazan, and the absorbance of each well was read at 570 nm using an automatic microplate reader (Molecular Devices Inc., Sunnyvale, CA, USA).

### Determination of caspase activity

Caspase activities were determined using a colorimetric assay kit (Calbiochem, Bad Soden, Germany) according to the manufacturer’s protocol. After treatment with different concentrations of tranilast, the cells were harvested and lysed in lysis buffer for 5 min in an ice bath. Equal amount of protein (10 μg) obtained by centrifugation of lysates at 10,000×*g* for 10 min was incubated with 80 μL of assay buffer and 10 μL of the substrate (acetyl-Asp-Glu-Val-Asp p-nitroanilide for caspase 3 activity or acetyl-Leu-Glu-His-Asp p-nitroanilide for caspase 9 activity) at 37 °C for 2 h. The optical density of the reaction mixture was determined using spectrophotometer at 405 nm.

### 4, 6-Diamidino-2-phenylindole (DAPI) staining

Treated cells were washed twice with phosphate buffered saline (PBS), fixed for 20 min in 4% paraformaldehyde, and washed three times in PBS. The cells were then blocked in 4% normal goat serum in PBS containing 0.3% Triton X-100 for 60 min. After rinsing twice with PBS, the cells were counterstained with DAPI in mounting medium (Vector Laboratories, Burlingame, CA, USA). Cover slips were mounted on microscope slides, and images were obtained using an Olympus LG-PS2 microscope (Tokyo).

### Flow cytometry

Following the indicated treatments, cell apoptosis was measured using Annexin V and propidium iodide (PI) double staining. The cells were washed with PBS, suspended in 100 μL of binding buffer (10 mM N -2-hydroxyethylpiperazine- *N*-2-ethanesulfonic acid/NaOH, 140 mM NaCl, 2.5 mM CaCl_2_, pH 7.4), and stained with 5 μL of FITC-conjugated annexin V antibody and 5 μL of PI (50 μg/mL). The mixture was incubated for 15 min at room temperature in the dark and analyzed by flow cytometry (Becton Dickinson Co.).

### Cell apoptosis

The apoptotic induction was determined using a cell death detection ELISA kit (Sigma-Aldrich) following the indicated treatment, which measures the specific mono- and oligonucleosomes in the cytoplasmatic fraction of cell lysates. The assay is based on a quantitative sandwich-enzyme-immunoassay-principle using monoclonal anti-histone and anti-DNA peroxidase antibodies. The relative apoptosis rate was photometrically determined by measuring the peroxidase activity of the immunocomplex at 405 nm, according to the manufacturer’s protocol.

### Determination of reactive oxygen species (ROS)

ROS were detected by 2'-7'-dichlorodihydrofluorescein diacetate (DCF-DA) fluorescence using a colorimetric assay kit (Calbiochem, Bad Soden, Germany) according to the manufacturer’s protocol. Briefly, aliquots (500 μL) of the cell lysates were incubated in the absence (control) or presence of 5 μM FeSO_4_ and/or xanthones III or V (0.5, 1, and 2.5 μM) at 37 °C in a shaking water bath for 2 h. Then, 100 μL of 75 μM DCF was added to the samples and incubated for 30 min in the dark. Finally, the samples were centrifuged at 6,000 × g for 15 min. ROS were conventionally detected in the supernatants by fluorescent spectrometry at 488 nm (excitation wavelength) and 532 nm (emission wavelength) using a Perkin-Elmer spectrometer. Results are expressed as percentage of ROS formation in treated cells versus control cells.

### Mitochondrial analysis

Mitochondrial transmembrane potential was measured using a JC‐1 kit (Qian Chen Inc, Shanghai, China) according to the manufacturer's protocol and analyzed using a laser scanning Varioskan Flash (Thermo Fisher). Green and red fluorescence were analyzed at 590 and 530 nm, respectively, and the ratio of green/red was considered an indicator of mitochondrial transmembrane potential.

### Extraction of nuclear and whole cell lysates and western blot analysis

To obtain nuclear extracts, the treated cells were washed once with PBS, and pelleted by centrifugation. The cell pellets were resuspended in hypotonic buffer (10 mM HEPES, pH 7.9, 1.5 mM MgCl_2_, 10 mM KCl, 0.2 mM phenylmethylsulfonylfluoride, 0.5 mM dithiothreitol, and 10 μg/mL aprotinin), incubated on ice for 15 min, lysed by adding 0.1% Nonidet P-40 and vortexed vigorously for 10 s. After centrifugation of lysates at 12,000 × g for 1 min at 4 °C, the resulting nuclei pellets were resuspended in high salt buffer (20 mM HEPES, pH 7.9, 25% glycerol, 400 mM KCl, 1.5 mM MgCl_2_, 0.2 mM EDTA, 0.5 mM dithiothreitol, 1 mM NaF, and 1 mM sodium orthovanadate), and used as nuclear fractions.

Separately, the washed cell pellets were lysed using extraction buffer PRO-PREP™ (Intron Biotechnology, Seongnam, Korea). After incubation for 30 min at 4 °C, the lysates were centrifuged to remove cell debris, and quantified using the Bio-Rad protein assay reagent according to the manufacturer's instructions. Equal amount of cellular protein (30 μg) from treated and untreated cell extracts were run onto the sodium dodecyl sulfate–polyacrylamide gel. After electrophoresis, proteins were transferred onto polyvinylidene difluoride membranes. After overnight blocking at 4 °C with 4% skim milk, the membranes were incubated with appropriate primary antibody (Santa Cruz Biotechnology) overnight. After several washes with Tween 20/Tris-buffered saline, the membranes were incubated with horseradish peroxidase-conjugated secondary antibody (Santa Cruz Biotechnology, 1:1000 silution) for 1 h at room temperature, washed three times with T-TBS and then developed using enhanced chemiluminescence (Amersham Life Science).

### FoxO-1 S256A mutant study

To prepare FoxO-1 S256A mutant (TCC → GCA) vector, site-specific mutagenesis was performed using a QuickChange site-directed mutagenesis kit (Stratagene) using following primers: PN-FKHR(FoxO-1)_S256A, 5’-AGAGCTGCAGCAATGGACAACAACAGTAAA-3’; PC-FKHR(FoxO-1)_S256A, 5’-GTTGTCCATTGCTGCAGCTCTTCTCCTAGG-3’. After mutagenesis, the sequence was verified by sequencing. For immunofluorescence detection of GFP-FoxO-1, CHO cells were plated onto slide glass and were transfected with a total of 1.0 μg of wild-type or mutated pcDNA GFP FKHR(FoxO-1)-S256A using FuGENE (3–4 μL per μg DNA; Roche). Then the cells were counterstained with DAPI to visualize the nuclei. After mounting, the sections were imaged with a Zeiss LSM 700 laser-scanning confocal microscope (Carl Zeiss) and analyzed with ZEN 2010 software (Carl Zeiss).

### RNA preparation and real-time qPCR

Total RNA was isolated from the cells using easy-Blue™ Kit (Intron Biotechnology). For each sample, 100 ng of RNA was reverse-transcribed (RT) using MuLV reverse transcriptase, 1 mM dNTP, and 0.5 µg/µL oligos (dT12-18). PCR amplification was performed using SYBR green. The PCR primers used were as follows: sense strand rPDX-1, 5′-AAATCC ACC AAA GCA CAC GC-3′, anti-sense strand rPDX-1, 5′-AAGTTGAGCACT ACT GCC AGC-3′; sense strand rMafA, 5′-AGGAGGAGGTCATCCGACTG-3′, anti-sense strand rMafA, 5′-CTTCTCGCTCTCCAGAATGTG-3′; sense strand rInsulin, 5′- GAC CCG CCAGTGCCACCA-3′, anti-sense strand rInsulin, 5′-TCCACAAGCCACGCATCTG-3′; sense strand rFoxO-1, 5′- AATTTGCTAAGAGCCGAGGA-3′; anti-sense strand rFoxO-1, 5′ -AAGTCATCATTGCTGTGGGA-3′; sense strand rDP, 5′-TCTGGAAGACACCCTCTGCT-3′, anti-sense strand rDP, 5′-CACAGAGTCCCACCATGTTG-3′; sense strand rGAPDH, 5′-GCCACTCAGAAGACTCTGGA-3′, anti-sense strand rGAPDH, 5′-GTTCAGCTCTGGGATGACCT-3′; sense strand rGRP78, 5′-CTG GAC TGA ATG TCA TGA GGA TCA-3′, anti-sense strand rGRP78, 5′-CTC TTA TCC AGG CCA TAT GCA ATA G-3′; sense rATF3, 5′-AAC TGG CTT CCT GTG CAC TT-3′, anti-sense strand rATF3, 5′-TGA GGC CAG CTA GGT CAT CT-3′; sense strand rCHOP, 5′-CCA CCA CAC CTG AAA GCA GAA-3′, anti-sense strand rCHOP, 5′-GGT GCC CCC AAT TTC ATC T-3′; sense strand mPDX-1, 5′-AAA TCC ACC AAA GCA CAC GC-3′, anti-sense strand mPDX-1, 5′-AAG TTG AGC ACT ACT GCC AGC-3′; sense strand mInsulin, 5′-AAA TCC ACC AAA GCA CAC GC-3′, anti-sense strand mInsulin, 5′-AAG TTG AGC ACT ACT GCC AGC-3′; sense strand mFoxO-1, 5′-AAA TCC ACC AAA GCA CAC GC-3′, anti-sense strand, mFoxO-1, 5′-AAG TTG AGC ACT ACT GCC AGC-3′; sense strand mGAPDH, 5′- CTC AAC TAC ATG GTC TAC ATG TTC CA -3′, anti-sense strand mGAPDH, 5′- CCA TTC TCG GCC TTG ACT GT ′. The results are expressed as a ratio of β-actin expression.

### Isolation and culture of islets

Male C57BL/6J mice (8 weeks old) were injected with collagenase in the common bile duct under anesthesia, and the pancreas was isolated, minced, and placed in ice-cold Hanks solution. After incubation of the mixture at 37 °C for 15 min, the islets were hand-picked under a stereomicroscope, and cultured in RPMI medium supplemented with 10% heat-inactivated FBS, 25 mg/mL amphotericin B, 10,000 U/mL penicillin, and 10,000 mg/mL streptomycin at 37 °C in a humidified 5% CO_2_ atmosphere.

### Glucose stimulated insulin secretion (GSIS)

INS-1 cells or islets were plated in 24-well plates (5 × 10^4^ cells/well) and incubated with tranilast (10, 50, and 100 μM) in the presence or absence of 500 μM PA for 24 h, and the GSIS assay was performed as described previously^39^.

### Animals

Male C57BL/6J mice (6 weeks of age) were purchased from Orient Bio Inc. (Seongnam, Korea). C57BL/6J mice were acclimated for one week under constant conditions (temperature: 23 ± 2 °C, humidity: 40%–60%, light/dark cycle: 12 h). For the experiments, the mice were divided into three groups (n = 8 each group): (1) control group (treated with vehicle solution containing 0.5% carboxymethyl cellulose (CMC)); (2) tranilast (50 mg/kg)-treated group; (3) tranilast (100 mg/kg)-treated group. Each treatment was done by oral injection at three time points (9 AM, 7 PM, and 9 AM on the 2nd day).

Db/db mice (6 weeks old, Orient Bio Inc.) were acclimated to the constant conditions (temperature: 23 ± 2 °C, humidity: 40%–60%, light/dark cycle: 12 h) for one week prior to the experiments and maintained for 4 weeks or more. For the experiments, the mice were randomly assigned into four groups (n = 8 each group), and treated with (1) vehicle solution containing 0.5% CMC (control group), (2) tranilast (50 mg/kg in 0.5% CMC), (3) tranilast (100 mg/kg in 0.5% CMC), (4) tranilast (200 mg/kg in 0.5% CMC) by oral injection once daily for 4 weeks or more. Body weight, food intake, and blood glucose were monitored weekly at the same time every week (between 10:00 and 11:00 AM). The amount difference between the preweighed original amount of food and the weight of the food left in each cage (n = 4 per cage) were considered as food intake. Blood was obtained from the tail vein of the animals in a fed state, and glucose concentrations were determined using Gluco Dr. Plus (Allmedicus, Seoul, Korea). Animals were minimally anesthetized with 1–1.5% isoflurane using small animal anesthesia system, and tissues were rapidly isolated and weighed. Pancreatic tissues were fixed in 4% paraformaldehyde and stored at 25 °C until further analyses. Serum insulin levels were determined using ELISA kits (Becton Dickinson, Franklin Lakes, NJ, USA). All animal procedures were carried out in accordance with the Guide for the Care and Use of Laboratory Animals, published by the US National Institutes of Health (NIH Publication No. 85-23, revised 2011) and were approved by the Animal Care and Use Committee of Gachon University, Seongnam, Korea (approval No. LCDI-2018-0040, LCDI-2018-0067). All methods involving animals are reported in accordance with ARRIVE (Animal Research: Reporting of in Vivo Experiments) guidelines.

### Glucose tolerance test

For glucose tolerance test, the mice were orally given glucose (2 g/kg), and blood was collected at 0, 30, 60, and 120 min after glucose administration from the tail vein into capillary tubes (Chase Scientific Glass). Plasma glucose levels were measured using Gluco Dr. Plus (Allmedicus). The area under the curve (AUC) for glucose was calculated using OriginPro 6.1 (Origin, Northampton, MA, USA).

### Histopathology

Pancreatic tissues fixed in 4% paraformaldehyde were embedded in paraffin, cut into 7 μm thick sections, and incubated with monoclonal antibodies against pFoxO-1, FoxO-1, PDX-1, and insulin (Santa Cruz Biotechnology) overnight at 4 °C. Then the sections were treated with biotin-conjugated goat anti-rabbit secondary antibody (Vector Laboratories, Burlingame, CA, USA; diluted 1:250), followed by the treatment with streptavidin-conjugated with horseradish peroxidase (Vector Laboratories, diluted 1:250). DAB peroxidase substrate (Vector Laboratories) was used for visualization, and the specimens were counterstained with hematoxylin (Sigma Chemical Co.).

### Statistical analysis

Data were analyzed using Student’s *t*-test for paired experiments or two-way analysis of variance. Results are expressed as the mean ± standard deviation (SD) of three separate experiments, and *p* < 0.05 was considered statistically significant.

## Supplementary Information

Supplementary Information.

## Data Availability

The datasets and reagents generated during the current study are available from the corresponding author on reasonable request or are present in the supplementary information.
