# Supplementary figures and images for: Tranilast protects pancreatic β-cells from palmitic acid-induced lipotoxicity via FoxO-1 inhibition
Source: Sci Rep. 2023 Jan 3;13:101. doi: 10.1038/s41598-022-25428-3 (PMC9810694; doi:10.1038/s41598-022-25428-3)

Figure 2B

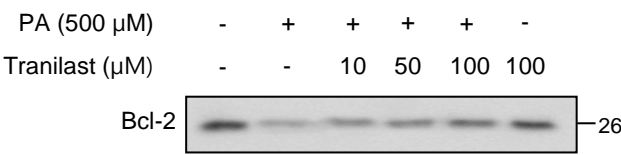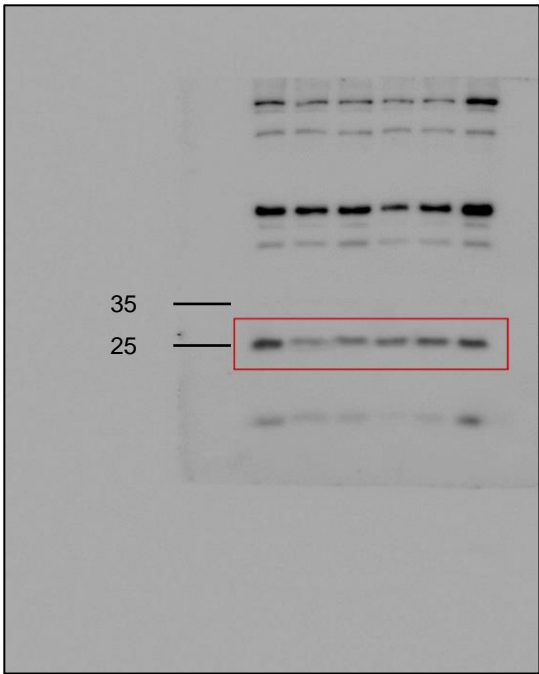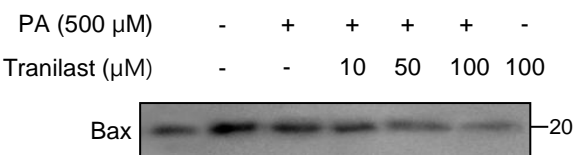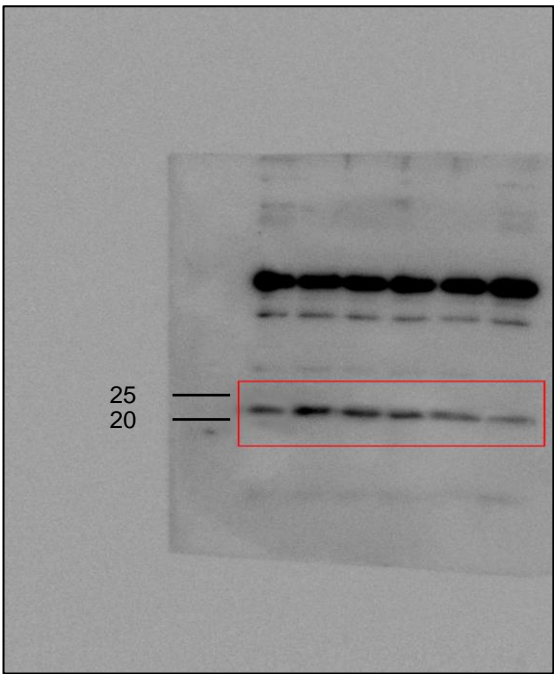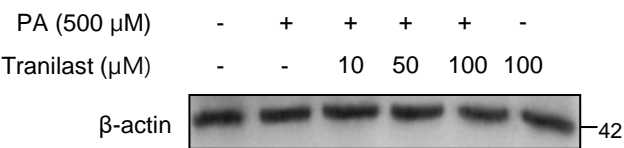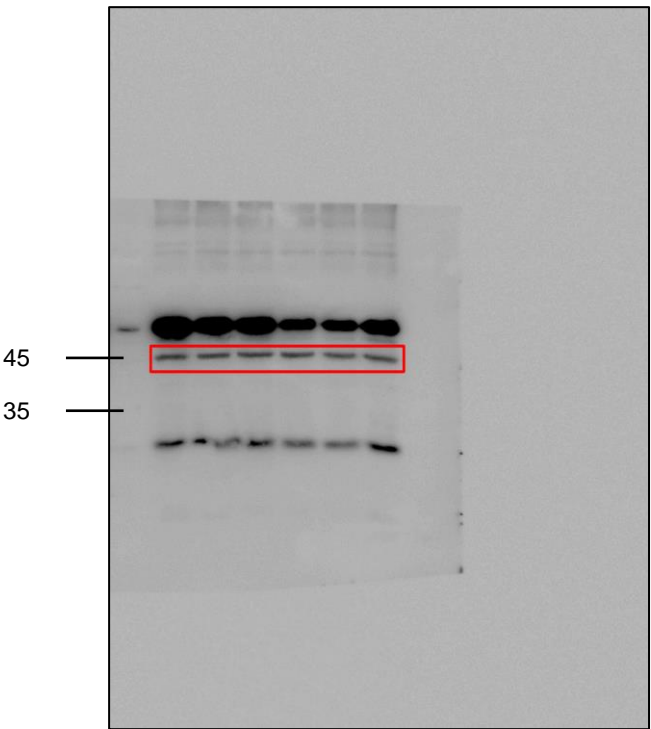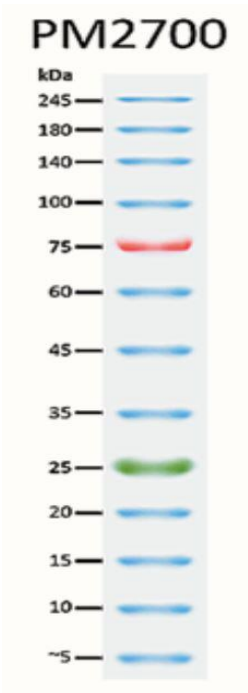

Figure 4B

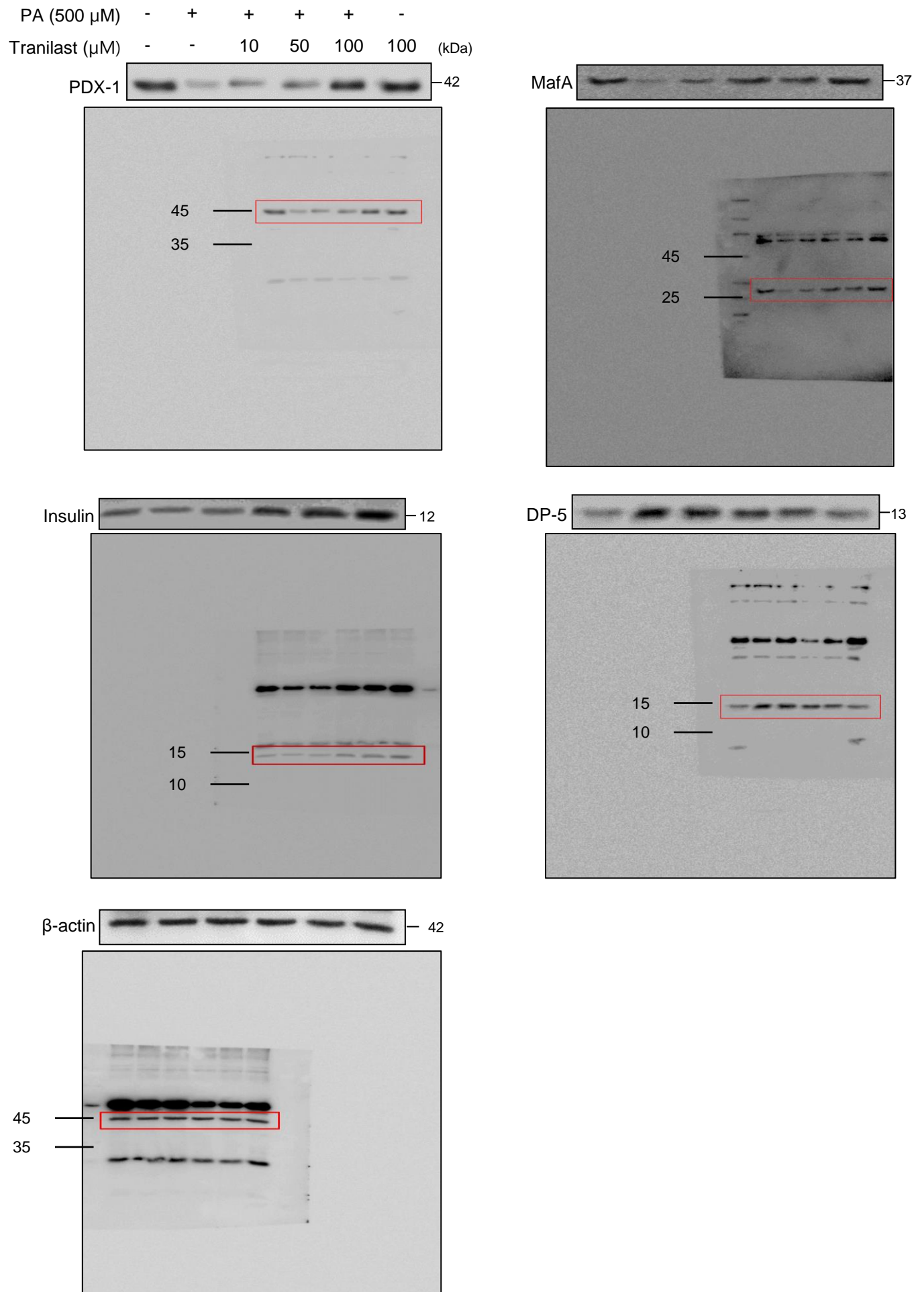

Figure 5A

|                      |   |   |    |    |     |     |
|----------------------|---|---|----|----|-----|-----|
| PA (500 $\mu$ M)     | - | + | +  | +  | +   | -   |
| Tranilast ( $\mu$ M) | - | - | 10 | 50 | 100 | 100 |

|         |                                                                                    |    |
|---------|------------------------------------------------------------------------------------|----|
| pFoxO-1 | 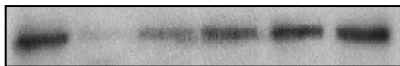  | 82 |
| FoxO-1  | 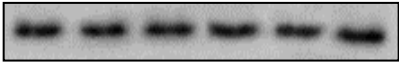 | 82 |

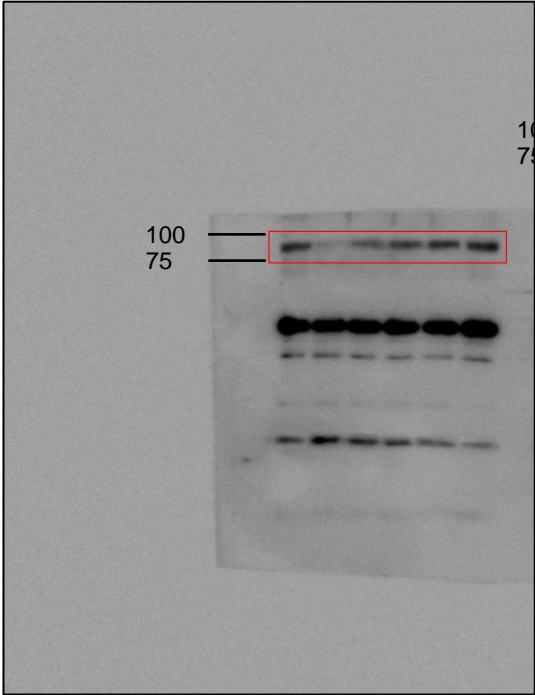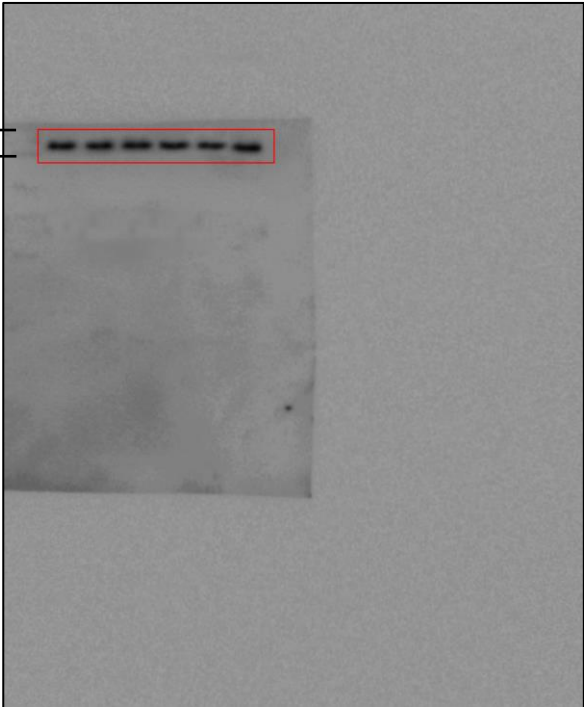

|                |                                                                                     |    |
|----------------|-------------------------------------------------------------------------------------|----|
| $\beta$ -actin | 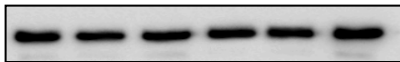 | 42 |
|----------------|-------------------------------------------------------------------------------------|----|

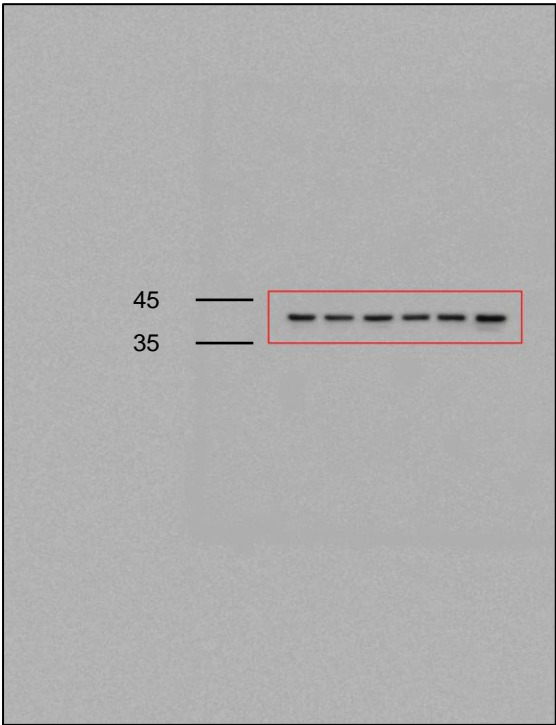

Figure 5B

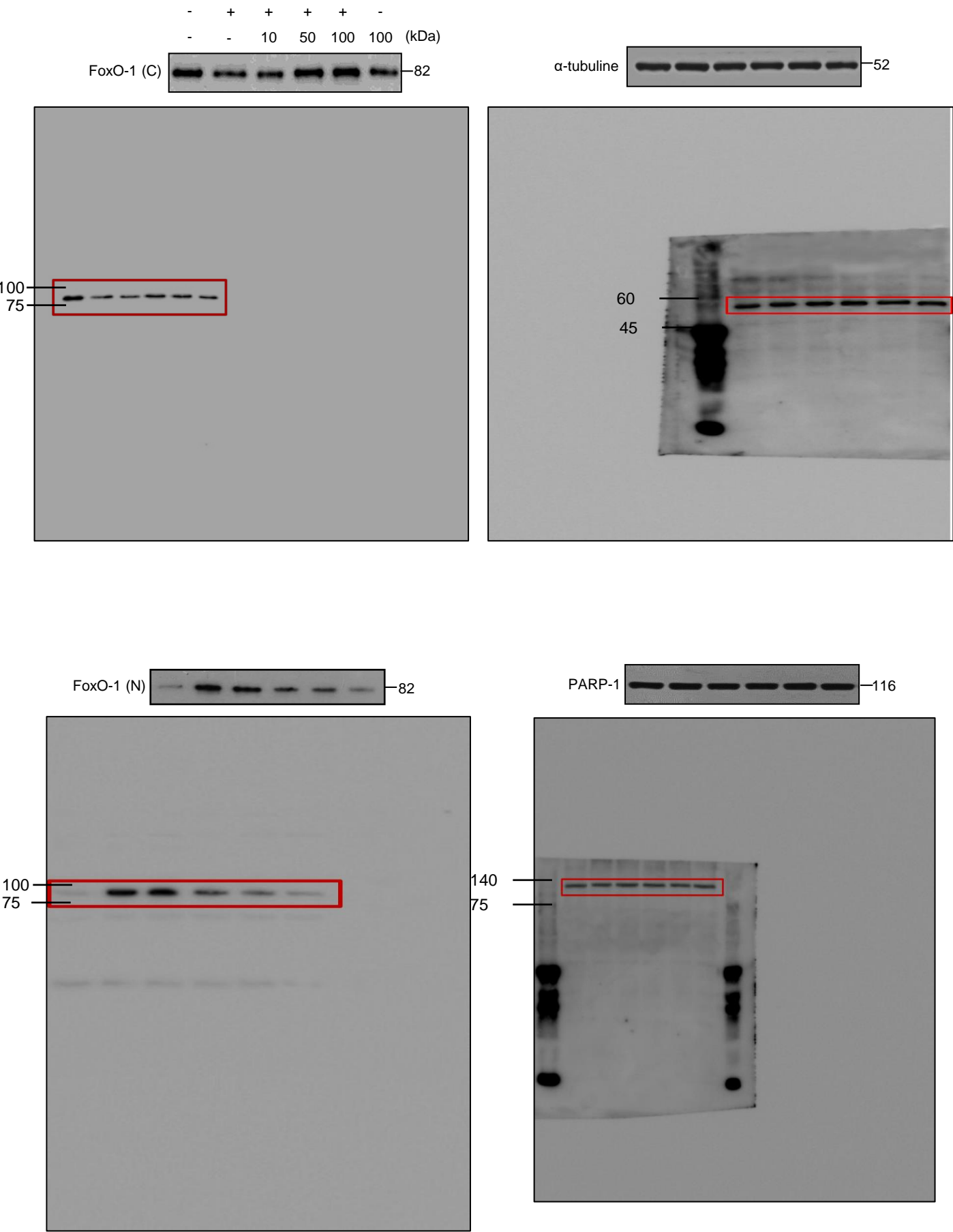

Figure 6D

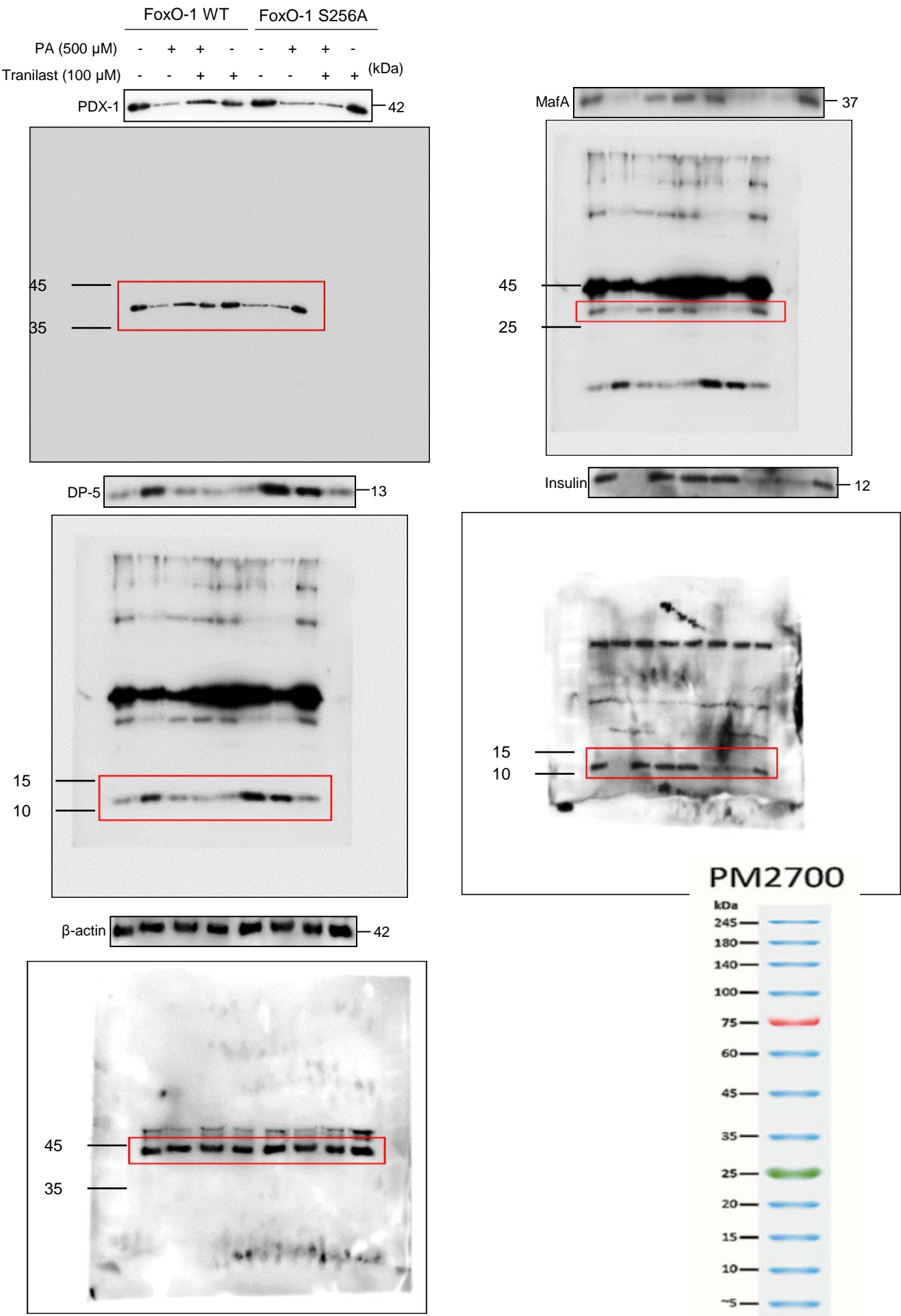

Supplement: Supplementary file 1 — Supplementary Information. [file 41598_2022_25428_MOESM1_ESM.pdf]
